# Treatment Approaches in 102 Elderly Patients With Non-Small Cell Lung Cancer

**DOI:** 10.14740/wjon894w

**Published:** 2015-02-14

**Authors:** Sener Cihan, Hatice Odabas, Nuriye Yildirim Ozdemir, Dogan Yazilitas, Nalan Akgul Babacan

**Affiliations:** a Department of Medical Oncology, Okmeydani Training and Research Hospital, 34100 Sisli, Istanbul, Turkey; b Department of Medical Oncology, Dr. Lutfi Kirdar Kartal Education and Research Hospital, 34860 Kartal, Istanbul, Turkey; c Department of Medical Oncology, Ankara Numune Training and Research Hospital, 06100 Altindag, Ankara, Turkey; d Department of Medical Oncology, Marmara University Pendik Education and Research Hospital, 34860 Kartal, Istanbul, Turkey

**Keywords:** Middle-old patient, Non-small lung cancer, Treatment, Survival

## Abstract

**Background:**

The life expectancy and presence of co-morbidities cause reservations in treatment decisions for elderly patients with cancer. In this study, we retrospectively evaluated 102 patients who are considered as middle-old aged (aged 75 - 84) by gerontologists.

**Methods:**

Medical records of patients were reviewed. One hundred and two patients with a diagnosis of non-small cell lung cancer (NSCLC) whose follow-up ended with death between March 2006 and May 2013 were examined.

**Results:**

The median age at diagnosis was 77 (75 - 85) years. Thirty-three patients (67.6%) were over 80 years old. The number of patients with metastasis was 57 (55.8%). Forty-two (41.2%) patients had stage IIIA and IIIB disease. Fifteen of the metastatic patients (26.3%) were given chemotherapy, while 12 of the non-metastatic patients (26.6%) were given chemotherapy. Of the non-metastatic patients, 25 (55.6%) were treated with radiotherapy, and five (11.1%) were treated with chemotherapy. The median duration of follow-up was 4 (1-55) months. Progression-free survival (PFS) was 4 months in non-metastatic patients, and 3 months in metastatic patients. Overall survival (OS) was 4 months. OS rates for 1 and 2 years were 10% and 2%.

**Conclusion:**

Chemotherapy and radiotherapy may be administered even to patients of this age group. The beneficial effect of chemotherapy in patients with metastasis on OS is an important finding of our study.

## Introduction

The risk of cancer increases with aging. The median age of lung cancer patients is 70 and thus it is a type of cancer that is more often seen in the elderly [1]. In the USA, 47% of all patients with non-small cell lung cancer (NSCLC) are in the age group of 70 years and older. Of 1,200,000 patients with NSCLC worldwide, 300,000 are over 70 years of age [2, 3]. Multi-organ failure, multiple drug use, and a decrease in the functional reserve of organs such as bone marrow are seen with aging. This makes cancer treatment more important and difficult in the elderly. Only a quarter of the patients with NSCLC at the age of 65 years and older could be given chemotherapy in the study by Earle et al [4]. Life expectancy in the elderly and side effects of treatments are important factors in cancer therapy. Although ECOG performance scoring may be used to show the performance in many cases, activities of daily living (ADL) and instrumental activities of daily living (IADL) performance systems are more convenient in the elderly [5]. The comprehensive geriatric assessment (CGA) is another scoring system used in chemotherapy or palliative therapy of elderly patients [6-9].

Cisplatin-based chemotherapeutic regimens are accepted generally for younger patients [10]. There are many studies in the literature on chemotherapy in elderly patients and on therapeutic regimens [11-16].

Chronologically, old is considered as an age of 65 years and over. The World Health Organization has defined the psychogeriatric aging as “old” for those at an age of 65 years and over, and “very old” for those at an age of 85 and over. On the other hand, according to the classification of gerontologists, those between 65 and 74 years are defined as young-old aged, those between 75 and 84 years are middle-old aged and those over 85 years are defined as the oldest-old [17, 18]. We present here a retrospective evaluation of 102 patients in the middle-old age (aged 75 - 84) with NSCLC.

## Methods

Medical records of patients followed up at two different oncology centers were reviewed. A total of 102 patients with NSCLC who were followed up between March 2006 and May 2013, and had eventually died were examined.

Survival measures were progression-free survival (PFS) and overall survival (OS), as most of the patients were stage III and stage IV. PFS was determined as time to local recurrence, metastasis, death without progression of disease or last control from diagnosis. OS was determined as time to death or last control. Both of these were calculated as the mean number of months. Statistical analyses were done with Statistical Package for Social Sciences (SPSS 20.0) software. Survival analyses were done according to the Kaplan-Meier method, and Fisher’s exact test and Chi-square tests were used to evaluate nominal variables and numeric data.

## Results

Clinical and pathological characteristics of patients are summarized in Table 1. The median age at diagnosis was 77 (75 - 85) years. Thirty-three patients (67.6%) were over 80 years. Although stage IV disease was more prevalent in both groups, the difference was not statistically significant (P = 0.77). Only four (3.9%) of the patients were females. All female patients had the adenocarcinoma subtype with bone-metastatic stage IV disease. Sixteen (88.9%) patients with a hemoglobin value below 10 mg/dL at diagnosis had stage IV disease and this finding was statistically significant (P = 0.02).

**Table 1:** Patient Characteristics

Characteristics	No treatment group, n (%)	Treatment group, n (%)	P value	Total, n (%)
Median age (min - max)	78 (75 - 85)	76 (75 - 85)	0.36	77 (75 - 85)
Gender			0.41	
Male	53 (94.6)	45 (97.8)		98 (96.1)
Female	3 (5.4)	1 (2.2)		4 (3.9)
Age			0.42	
< 80	36 (64.3)	33 (71.7)		69 (67.6)
> 80	20 (35.7)	13 (28.3)		33 (32.4)
Surgery			0.53	
None	55 (98.2)	44 (95.6)		99 (97)
Lobectomy	1 (1.8)	1 (2.2)		2 (2)
Pneumonectomy	0	1 (2.2)		1 (1)
Histological type				
Adenocancer	15 (26.8)	5 (10.8)	0.048	20 (19.6)
Squamous cancer	27 (48.2)	31 (67.4)		58 (56.9)
Unknown	14 (25)	10 (21.8)		24 (23.5)
Hemoglobin in diagnosis				
< 10	19 (33.9)	7 (15.2)	0.003	26 (25.5)
> 10	37 (66.1)	39 (84.8)		76 (74.5)
Baseline co-morbidities				
Hypertension	13 (23.2)	13 (28.3)	0.005	26 (25.5)
Cardiac co-morbidities	14 (25)	11 (23.9)		25 (24.5)
Pulmonary co-morbidities	6 (10.7)	4 (8.7)		10 (9.8)
Diabetes mellitus	2 (3.6)	10 (21.8)		12 (11.8)
≥ 3 co-morbidities	15 (26.8)	2 (4.3)		17 (16.7)
ECOG performance status				
0	2 (3.6)	3 (6.5)	< 0.001	5 (4.9)
1	4 (7.2)	21 (45.6)		25 (24.5)
2	13 (23.2)	13 (28.3)		26 (25.5)
≥ 3	37 (66)	9 (19.6)		46 (45.1)
Stage				
1	0	2 (4.3)	< 0.001	2 (2)
2	1 (1.8)	0		1 (1)
3A	3 (5.4)	8 (17.4)		11 (10.8)
3B	10 (17.8)	21 (45.6)		31 (30.4)
4	42 (75)	15 (32.7)		57 (55.8)
Metastasis site				
Brain	6 (10.7)	2 (4.3)	0.003	8 (14)
Adrenal glands	1 (1.8)	1 (2.2)		2 (3.5)
Bone	4 (7.2)	5 (10.9)		9 (15.8)
Contralateral lung	10 (17.8)	3 (6.5)		13 (22.8)
Multiple metastasis	21 (37.5)	4 (8.7)		25 (43.9)
Treated with chemotherapy				
Metastatic disease	N	N		15 (26.3)
Locally advanced disease				12 (26.6)
No				73 (71.6)
Treated with radiotherapy				
Radiotherapy	N	N		25 (55.6)
Chemoradiotherapy				5 (11.1)
Palliative radiotherapy				39 (68.2)

Low hemoglobin value was found to be a factor with an effect on OS (P < 0.001). Only three patients (2.9%) were treated surgically (one pneumonectomy and two lobectomies). The patient who had undergone pneumonectomy was 75 years old and had stage IIIA disease. Four courses of adjuvant cisplatin/etoposide were administered and the patient survived for 28 months, which is considerably longer than OS. In the pathological subtyping, 20 patients (19.6%) had adenocancer, 58 (56.9%) had squamous cell carcinoma, whereas subtyping could not be done in 24 patients (23.5%). A significant effect of the pathological subtypes on PFS (P = 0.99) and OS (P = 0.59) was not detected. A total of 88 patients (86.3%) had inoperable disease, with stage IIIB disease in 31 patients (30.4%) and stage IV disease in 57 patients (55.8%). Patients with multiple metastases, including to the brain, had a shorter OS (median 2 months), while the best OS was detected in patients with contralateral lung metastasis or bone metastasis (median 6 months).

Chemotherapy was administered to 15 (26.3%) of the metastatic patients, while 39 (68.2%) were administered palliative radiotherapy. There was a statistically significant OS difference between metastatic patients who were administered chemotherapy, with an OS median of 6 months (95% CI: 4.1 - 7.9 months) and patients who were not administered chemotherapy, with an OS median of 3 months (95% CI: 2.3 - 3.7 months) (P = 0.02). Twelve of the patients with non-metastatic disease (26.6%) were administered only chemotherapy, while five (11.1%) were administered chemoradiotherapy and 25 (55.6%) only radiotherapy. The worst survival was in the chemoradiotherapy arm, with a median of 4 months, while survival had a median of 9 months in the radiotherapy arm and a median of 10 months in the chemotherapy arm. But these values were not statistically significant (P = 0.53). In the comparison of the group which was administered one of these three treatment modalities and those who did not receive any treatment in the non-metastatic patient group, the OS in patients who were treated was better, with a median of 8 months (95% CI: 5.1 - 10.9) compared to a median of 4 months without treatment (95% CI: 1.4 - 6.6). But this difference did not reach statistical significance (P = 0.12).

**Figure 1:**
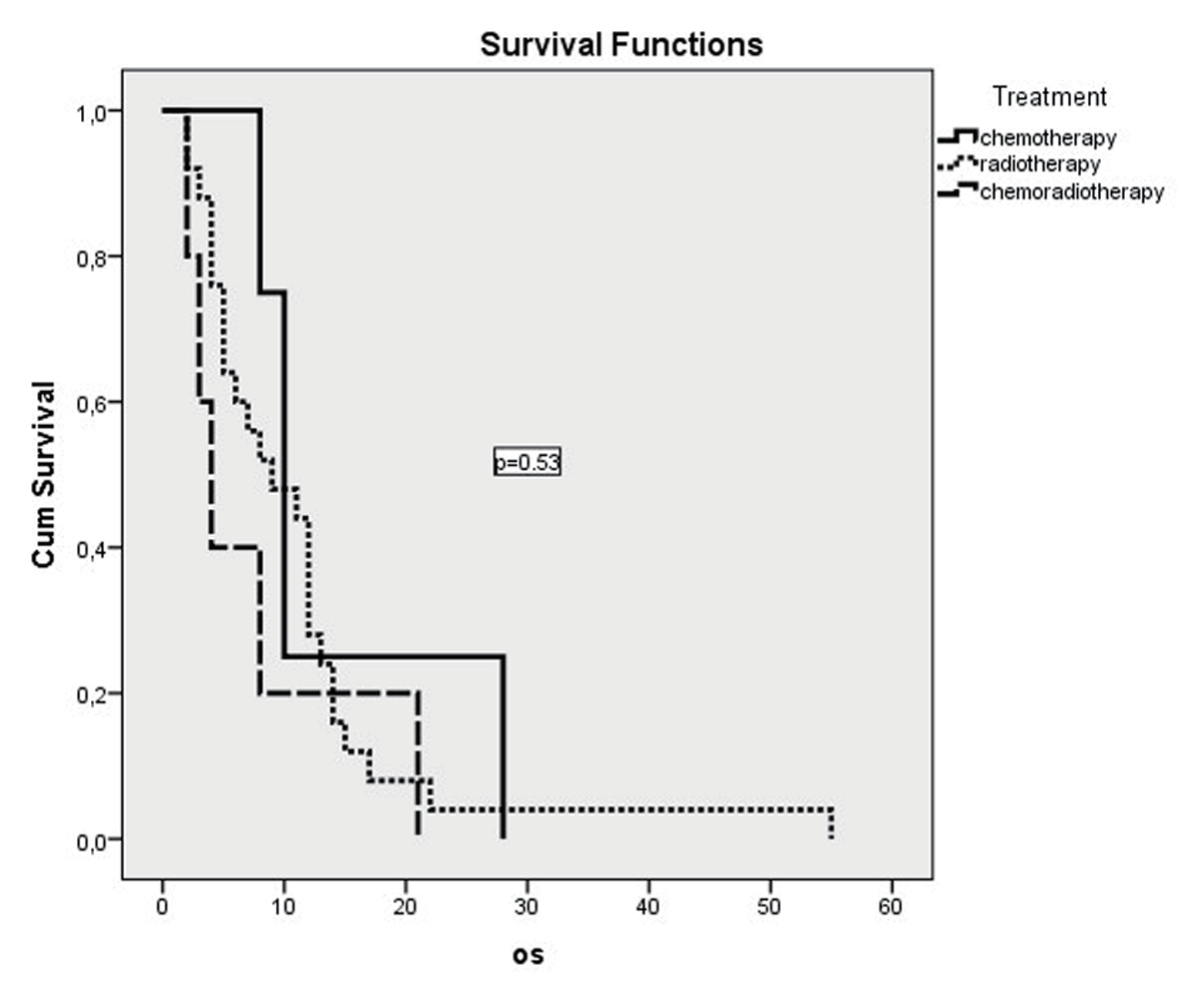
OS curves of non-metastatic patients who had received treatment.

**Figure 2:**
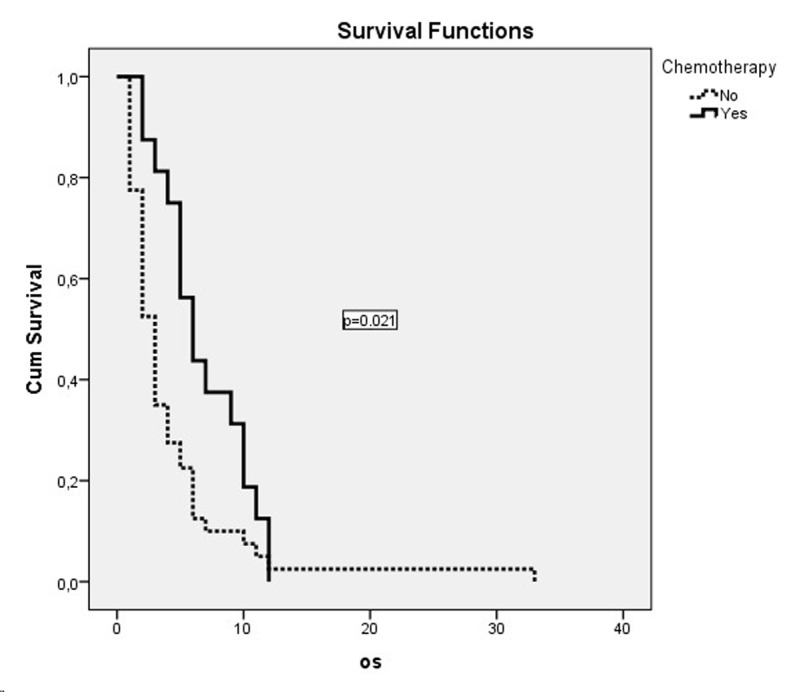
OS curves of metastatic patients who had received chemotherapy.

Dual combination chemotherapy regimens were used in both metastatic and non-metastatic patient groups (Table 2). The gemcitabine/cisplatin combination was the most frequently used combination in patients with metastatic disease. But this combination did not have a significant effect on survival in comparison with other combinations (P = 0.65). The second most frequently used combination was the docetaxel/cisplatin combination. A significant effect of gemcitabine or taxane use on survival was not detected (P = 0.54). The cisplatin or carboplatin combinations did not show a significant effect on survival in both metastatic and non-metastatic patients. The survival curves of treatment modalities in non-metastatic and metastatic patients are shown in Figure 1 and Figure 2, respectively.

**Table 2:** Chemotherapy Regimens Used in Metastatic and Non-Metastatic Setting

Chemotherapy regimens	Number of patients, n (%)		Explanation
	Non-metastatic	Metastatic	
Gemcitabine/cisplatin	2 (16.7)	6 (40)	Gemcitabine 1,000 mg/m^2^ day 1 and 8; cisplatin 60 mg/m^2^ day 1 every 21 days
Docetaxel/cisplatin	1 (8.3)	3 (20)	Docetaxel 60 mg/m^2^ day 1; cisplatin 60 mg/m^2^ day 1 every 21 days
Gemcitabine/carboplatin	2 (16.7)	1 (6.7)	Gemcitabine 1,000 mg/m^2^ day 1 and 8; carboplatin AUC 4 day 1 every 21 days
Docetaxel/carboplatin	1 (8.3)	2 (13.3)	Docetaxel 60 mg/m^2^ day 1; carboplatin AUC 4 day 1 every 21 days
Vinorelbine/cisplatin	1 (8.3)	2 (13.3)	Vinorelbine 25 mg/m^2^ day 1 and 8; cisplatin 60 mg/m^2^ day 1 every 21 days
Vinorelbine	1 (8.3)	1 (6.7)	Vinorelbine 25 mg/m^2^ IV first day and 60 - 80 mg/m^2^ oral day 1 and 8 every 21 days
Gemcitabine	1 (8.3)	No	Gemcitabine 1,200 mg/m^2^ days 1 and 8 every 21 days
Cisplatin/etoposide	3 (25)	No	Cisplatin 60 mg/m^2^ day 1; etoposide 100 mg/m^2^ days 1, 2 and 3 every 21 days

The median follow-up period was 4 (1 - 55) months. PFS was 4 months (95% CI: 2.1 - 5.9 months) in non-metastatic patients and 3 months (95% CI: 2.6 - 3.3 months) in metastatic patients. OS was 4 months (95% CI: 2.9 - 5.1 months). OS rates for 1 and 2 years were 10% and 2%. Parameters that might have an effect on survival are summarized in Table 3.

**Table 3:** P values of Prognostic Factors

Factor	P value	
	Progression-free survival	Overall survival
Gender	0.802	0.635
Hemoglobin	0.903	< 0.001
Pathology	0.986	0.592
LVI	0.031	0.135
Perineural invasion	0.072	0.342
Stage	0.515	< 0.001
Metastatic site	0.032	< 0.001
Receiving chemotherapy	0.094	0.021
Receiving treatment	0.671	0.534

## Discussion

Gerontologists classify aging as young-old age between 65 and 74 years, middle-old age between 75 and 84 years, and oldest old age above 85 years [17, 18]. Life expectancy is limited in this age group. The question whether a patient will die from cancer or from other natural causes should be answered. Another problem is the side effects related to treatment, which may cause deterioration of the present condition of the patient during therapy. ADL, IADL [5], and the CGA [6-9] are systems developed to predict the performance and chemotherapy tolerance of elderly patients. The most appropriate patients may be selected with the help of these systems.

There are studies showing the absence of a difference in survival of elderly (> 70 years) and younger patients with stage I, II and some selected IIIA NSCLC, who received surgical treatment [19, 20]. Gray et al have shown a positive effect of surgical treatment on survival in patients older than 66 years with NSCLC [21]. We did not observe a significant difference in OS in the three patients who were surgically treated in our study (P = 0.28). This finding may be explained by the limited number of surgical patients in our study. On the other hand, an OS of 28 months observed in the 75-year-old patient with stage IIIA disease who had received pneumonectomy and adjuvant chemotherapy is an important finding. Although surgical treatment is controversial in the medical literature [20, 22], surgery should be considered in appropriate middle-aged patients.

Adjuvant chemotherapy is controversial. Pepe et al have reported a positive effect of chemotherapy on OS in patients aged > 65 years, and a negative effect on OS in patients aged > 75 years [23]. Patients over 70 years who were administered chemotherapy were reported to be able to receive lower doses of cisplatin, also with fewer chemotherapy cycles [24].

As in our study, most of the old patients are not candidates for surgery, due to the presence of co-morbidities or advanced stage disease. There are studies that have shown the feasibility of definitive radiotherapy or sequential radiotherapy with chemotherapy in such patients [25-28]. Although chemoradiotherapy is more toxic in comparison to younger patients, it may also be used in elderly patients [29-32]. But caution is required for patient selection for chemoradiotherapy. In our study, although not statistically significant, the shortest survival was observed in patients who had received chemoradiotherapy, which was 4 months (95% CI: 1.8 - 6.1 months, P = 0.7).

Most of the elderly patients are diagnosed at metastatic stages. The ratio of metastatic disease was 55.8% in our study. Both the short OS in metastatic NSCLC and the short life expectancy in this age group cause reservations for chemotherapy of metastatic patients. But there are studies reporting improvement in survival with vinorelbin as a single agent in comparison with BSC in patients older than 70 years with metastatic NSCLC, and a decrease in complaints due to the tumor [33]. Later, positive effects of docetaxel and paclitaxel in this patient group were also reported [34].

Should chemotherapy regimens contain a single agent or dual agents? In a phase III study, Quoix et al administered a weekly dual regimen including paxlitaxel and carboplatin to patients 70 - 89 years of age, and vinorelbin or gemcitabine as a single agent to other groups [35]. The median OS was 10.3 months in the dual agent chemotherapy group and 6.2 months in the single agent group, with a statistically significant difference (P < 0.001). As a result of this study, a combination regimen including weekly carboplatin and paclitaxel in middle-old age patients was recommended as first line treatment. Langer et al reported that chemotherapy regimens including cisplatin may be used even in elderly patients [36]. Our study seemingly indicates that combination chemotherapy regimens were preferred. The number of courses was between 2 and 6. Toxicity and side effects were also more frequently encountered. In our study, chemotherapy administered to metastatic patients was found to be a factor that has a statistically significant effect on survival (P < 0.04).

The use of cisplatin or carboplatin in dual chemotherapy regimens in this age group is important. There are studies in the medical literature that have shown no difference between cisplatin and carboplatin on OS when administered to patients over 70 years of age [37, 38]. Blanchard et al have reported negative effects of cisplatin use in elderly patients [39]. Our study was conducted with patients in the 75 - 85 year old group. No difference between the effects of cisplatin or carboplatin use on OS was detected in these patients (P = 0.39).

Old patients represent a special population. An elderly patient with metastatic lung cancer may be considered as “already doomed for death”, but as was the case in our study with the 80-year-old patient with stage IIIB NSCLC, a survival of 55 months may be obtained with an appropriate treatment after a sound evaluation and appropriate patient selection.
